# CTSE Overexpression Is an Adverse Prognostic Factor for Survival among Rectal Cancer Patients Receiving CCRT

**DOI:** 10.3390/life11070646

**Published:** 2021-07-02

**Authors:** Chia-Lin Chou, Tzu-Ju Chen, Yu-Feng Tian, Ti-Chun Chan, Cheng-Fa Yeh, Wan-Shan Li, Hsin-Hwa Tsai, Chien-Feng Li, Hong-Yue Lai

**Affiliations:** 1 Division of Colon and Rectal Surgery, Department of Surgery, Chi Mei Medical Center, Tainan 710, Taiwan; 991101@mail.chimei.org.tw (C.-L.C.); d870722@mail.chimei.org.tw (Y.-F.T.); 2 Department of Clinical Pathology, Chi Mei Medical Center, Tainan 710, Taiwan; a108n2@mail.chimei.org.tw (T.-J.C.); livelychord.tsai@biocheck.com.tw (H.-H.T.); 3 Department of Medical Technology, Chung Hwa University of Medical Technology, Tainan 717, Taiwan; a80818@mail.chimei.org.tw; 4 Institute of Biomedical Sciences, National Sun Yat-Sen University, Kaohsiung 804, Taiwan; 5 Department of Medical Research, Chi Mei Medical Center, Tainan 710, Taiwan; 090807@nhri.edu.tw; 6 National Institute of Cancer Research, National Health Research Institutes, Tainan 704, Taiwan; 7 Department of Internal Medicine, Chi Mei Medical Center, Tainan 710, Taiwan; 970402@mail.chimei.org.tw; 8 Institute of Precision Medicine, National Sun Yat-Sen University, Kaohsiung 804, Taiwan; 9 Department of Pathology, School of Medicine, College of Medicine, Kaohsiung Medical University, Kaohsiung 807, Taiwan

**Keywords:** rectal cancer, chemoradiotherapy, CTSE, digestion, immune response

## Abstract

The introduction of preoperative concurrent chemoradiotherapy (CCRT) increases the rate of anal preservation and allows tumor downstaging for clinical stage T3/T4 or node-positive rectal cancer patients. However, there is no precise predictive tool to verify the presence of residual tumor apart from surgical resection. The gastrointestinal (GI) tract not only digests nutrients but also coordinates immune responses. As the outermost layer of the GI tract, mucus plays a key role in mediating the interaction between the digestive and immune systems, and aberrant mucus mesh formation may cause chemoresistance by impeding drug delivery. However, the correlations among digestion-related genes, mucin synthesis, and chemoresistance remain poorly understood. In the present study, we evaluated genes related to digestion (GO: 0007586) and identified cathepsin E (*CTSE*), which is involved in immune regulation, as the most significantly upregulated gene associated with CCRT resistance in rectal cancer in a public transcriptome dataset (GSE35452). We recovered 172 records of rectal cancer patients receiving CCRT followed by surgical resection from our biobank and evaluated the expression level of CTSE using immunohistochemistry. The results revealed that tumors with CTSE overexpression were significantly correlated with pre-CCRT and post-CCRT positive nodal status (both *p* < 0.001), advanced pre-CCRT and post-CCRT tumor status (*p* < 0.001 and *p* = 0.002), perineural invasion (*p* = 0.023), vascular invasion (*p* < 0.001), and a lesser degree of tumor regression (*p* = 0.003). At the univariate level, CTSE overexpression was an adverse prognostic factor for all three endpoints: disease-specific survival (DSS), metastasis-free survival (MeFS) (both *p* < 0.0001), and local recurrence-free survival (LRFS) (*p* = 0.0001). At the multivariate level, CTSE overexpression remained an independent prognostic factor for poor DSS, MeFS (both *p* = 0.005), and LRFS (*p* = 0.019). Through bioinformatics analysis, we speculated that CTSE overexpression may confer CCRT resistance by forming a defensive mucous barrier. Taken together, these results suggest that CTSE overexpression is related to CCRT resistance and inferior survival in rectal cancer patients, highlighting the potential predictive and prognostic value of CTSE expression.

## 1. Introduction

Originating in the colon (large intestine) or rectum, colorectal cancer (CRC) affects the lower digestive system and has increasingly become a substantial global burden. The American Cancer Society (ACS) estimates that rectal cancer has accounted for approximately forty percent of the newly diagnosed CRC cases in 2021. It is noteworthy that Asia ranks first in terms of new CRC cases, CRC deaths, and the CRC 5-year prevalence rate [1]. In the early stage of rectal cancer, surgical resection is the standard intervention. For clinical stage T3/T4 or node-positive rectal cancer patients, the introduction of neoadjuvant concurrent chemoradiotherapy (CCRT) before surgery increases the rate of anal preservation and allows tumor downstaging. At present, the assessment of the response to CCRT still relies on clinical procedures, including imaging techniques, random biopsy under colonoscopy, and digital rectal examination. However, there is no precise predictive tool to verify the presence of residual tumor apart from surgical resection. The identification of genetic biomarkers has benefitted from the advancement of precision medicine and can guide treatment modality selection more precisely (causing surgery to no longer be the only choice) and improve quality of life.

The gastrointestinal (GI) tract not only digests nutrients but also coordinates immune responses. It must generate tolerance against the luminal microbiota without triggering an overt immune response while still protecting the intestinal mucosa from invading pathogens and potentially harmful dietary antigens. The single layer of intestinal epithelial cells differs in cellular composition and structure between the small intestine and colon. The epithelium is not a linear layer but composed of invaginations termed crypts. The mucus layer covers the epithelium and separates luminal microbes from direct contact with epithelial cells, and the mucus of this layer is mainly secreted by goblet cells. The small intestine is covered by a single mucus layer. However, the colon is covered by two mucus layers, which consist of the inner mucus layer close to the crypts and the outer mucus layer near the lumen [2]. Mucus functions at the junction of bacteria and host immune responses, especially in the colon, which contains the highest microbial density. The mucus layer is continuously replenished by goblet cells, which push the bacteria out toward the lumen and can slow bacterial penetration. In the colon, the goblet cells on the surface maintain the inner mucus layer by continuous mucus secretion, while the goblet cells around the crypts secrete mucus upon stimulation, such as endocytosis or acetylcholine exposure [3]. Mucus hypersecretion has been connected to bacterial overgrowth and the induction of inflammatory responses, which can promote cancer development [4]. In addition, aberrant mucin synthesis has also been suggested to create a barrier preventing drug access to targets, causing cancer cell chemoresistance [5]. However, the correlations among digestion-related genes, mucin synthesis, and chemoresistance remain poorly understood.

Cathepsin E (CTSE) is found mainly in the GI tract and is also found in the gallbladder, pancreas, and urinary bladder. The human *CTSE* gene, which maps to chromosome 1q32.1, encodes an aspartic endopeptidase. It is an intracellular proteinase that appears to not be involved in extracellular functions such as the digestion of dietary protein [6]. Instead, CTSE is located primarily in the endosome (GO: 0005768) and plays a role in antigen processing and the presentation of exogenous peptides via MHC class II (GO: 0019886) and mucosal protection. It has been reported that CTSE deficiency is correlated with atopic dermatitis [7]. In contrast, CTSE overexpression has been suggested to promote gastric cancer [8], pancreatic ductal adenocarcinoma [9], cervical adenocarcinoma [10], and lung carcinoma [11] development. However, the role of CTSE in CRC is still poorly understood. In this study, we aimed to elucidate the role of CTSE in response to preoperative CCRT and correlate CTSE expression with clinical outcomes in our well-characterized rectal cancer cohort.

## 2. Patients and Methods

### 2.1. Transcriptomic Profiling of a Public Rectal Cancer Dataset

To identify prospective genes related to the response to CCRT, a public transcriptome dataset (GSE35452) of 46 tumor specimens from rectal carcinoma patients treated with preoperative CCRT was utilized for data mining. Before CCRT, biopsy specimens were gathered during colonoscopic examination in this dataset. To quantify expression levels, we computerized the raw CEL files with the statistical software Nexus Expression 3 (BioDiscovery, El Segundo, CA, USA). All probes were analyzed without preselection. Based on the response to CCRT determined by clinical assessment, the samples were separated into “responders” and “nonresponders”, and a comparative analysis was performed. We focused on differentially expressed genes related to digestion (GO: 0007586) and further chose those with a *p*-value less than 0.01 and expression fold change > ±0.1 log2 ratio for further analysis.

### 2.2. Patient Eligibility and Enrollment

This study was approved by the Institutional Review Board of Chi Mei Medical Center (10302014) and was performed with a total of 172 records of rectal cancer patients who were routinely followed up between 1998 and 2004 and with formalin-fixed paraffin-embedded (FFPE) tissue samples in the biobank. The primary clinical stage was determined by colonoscopic biopsy, and no distant metastasis was observed by chest X-radiography and/or abdominopelvic CT. All patients received a total radiation dose of 45–50 Gy in 25 fractions over a period of five weeks concomitant with 24 hr continuous infusion of 5-fluorouracil (5-FU)-based chemotherapy before proctectomy. Adjuvant chemotherapy was given for patients presenting with a pre-CCRT or post-CCRT nodal status greater than N1 or a tumor status greater than T3. All patients were routinely tracked following diagnosis until death or the last follow-up.

### 2.3. Histopathological and Immunohistochemical Assessments

To obtain more reliable results, two independent pathologists (Wan-Shan Li and Chien-Feng Li) who were blinded to the clinical information of the patients performed pathological analysis of the tumor specimens. The pretreatment and posttreatment T and N stages were determined in agreement with the 7th American Joint Committee on Cancer (AJCC) TNM staging system. The tumor regression grade, which is predictive of the tumor response to CCRT, was evaluated in concordance with the description by Dworak et al. [12]. Immunohistochemical staining was conducted in accordance with our previous study [13], and staining was performed with an anti-CTSE antibody. The H-score was employed to interpret CTSE immunoreactivity and was quantified with the following equation: H-score = Σ*Pi* (*i* + 1), where *Pi* is the percentage of stained tumor cells for each intensity, ranging from 0% to 100%, and *i* is the intensity of staining (0 to 3+). The H-score was calculated and varied from 100 to 400 as determined by a combination of the intensity and percentage of positively stained tumor cells. Tumors with H-scores above or identical to the median of all scored cases were considered to have high CTSE expression.

### 2.4. Statistical Analysis

The chi-square (χ^2^) test was used to determine the associations between clinicopathological factors and CTSE expression. Survival curves were generated by the Kaplan-Meier method, and the log-rank test was used to assess and compare the time from the operation to death (or last seen alive) or recurrence (or the last date the patient was seen relapse-free). Variables with prognostic importance in the univariate analysis were included in the Cox proportional hazard model for multivariate analysis. All statistical analyses were performed utilizing SPSS software version 22.0 (IBM Corporation, Armonk, NY, USA), and two-tailed tests with *p* < 0.05 were considered statistically significant.

## 3. Results

### 3.1. CTSE Is Identified as the Most Significant Differentially Expressed Gene Related to Digestion

**Figure 1:**
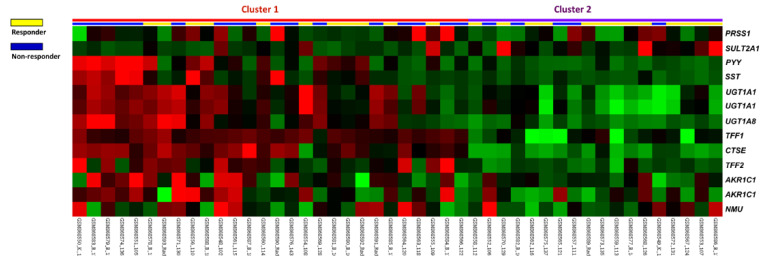
Transcriptomic profiling of genes associated with digestion and the response to CCRT. The expression levels of upregulated and downregulated genes were marked in red and green, respectively. The statistical significance of each transcript was examined by comparing responders to nonresponders. We identified *CTSE* as the most significantly upregulated gene linked to digestion (GO: 0007586) among patients unresponsive to CCRT.

To evaluate the efficacy of preoperative CCRT in rectal cancer patients, a public transcriptome dataset (GSE35452) including 46 rectal cancer patients was analyzed to find potential biomarkers. Twenty-four patients (52.2%) were categorized as responders, whereas 22 patients (47.8%) were classified as nonresponders in accordance with the response to CCRT. We identified 13 probes covering 11 transcripts focusing on digestion (GO: 0007586) (Table 1 and Figure 1). Among these genes, *CTSE* is most likely to be involved in immune regulation, and its expression was remarkably higher among CCRT nonresponders (log_2_ ratio = 1.7848, *p* < 0.0001). This encouraged us to further explore the expression status and clinical relevance of *CTSE* in rectal carcinoma.

### 3.2. Clinicopathological Characteristics of Rectal Carcinoma Patients in Our Cohort

A total of 172 records of rectal carcinoma patients receiving preoperative CCRT from 1998 to 2004 were retrieved from the biobank, and most patients were less than 70 years old (n = 106, 61.6%) and male (n = 108, 62.8%) (Table 2). During pre-CCRT clinical staging, the nodal status of 125 patients (72.7%) was negative (cN0), and the depth of invasion of 81 patients (47.1%) was limited to the muscularis propria (cT1-2). No local lymph node metastasis (ypN0) was observed in 123 patients (71.5%), and the invasive depth of 86 patients (50%) was pathologically limited to the muscularis propria (ypT1-2) following CCRT. No vascular invasion was observed in 157 (91.3%) patients, and no perineural invasion was detected in 167 (97.1%) patients. The tumor regression grade was utilized to predict the tumor response to CCRT, and the results revealed that 17 patients (9.9%) had a complete response (grade 4), 118 patients (68.6%) had a modest response (grade 2–3), and 37 patients (21.5%) had no or little response (grade 0–1).

### 3.3. CTSE Immunoexpression and Its Correlations with Clinicopathological Factors

**Figure 2:**
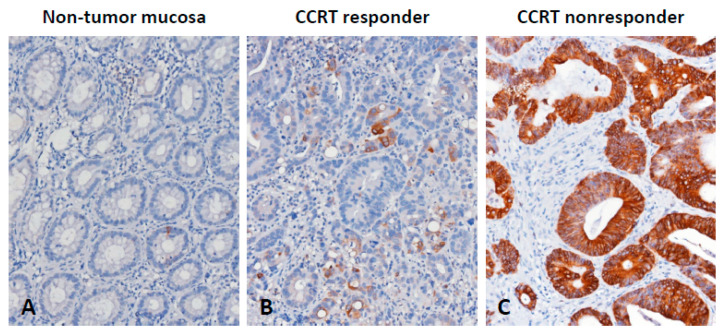
Immunohistochemical detection of CTSE. (**A**) Non-tumor mucosa presented no expression of CTSE. Rectal carcinoma samples showed (**B**) low expression of CTSE in patients responsive to CCRT and (**C**) high expression of CTSE in patients unresponsive to CCRT.

Immunohistochemical staining was employed to investigate the correlations between CTSE immunoexpression and its clinical relevance in rectal carcinoma (Table 2). As shown in Figure 2, CTSE immunoreactivity in CCRT-responsive rectal carcinoma tissue specimens was significantly lower than that in CCRT-nonresponsive rectal carcinoma tissue specimens. CTSE overexpression was remarkably correlated with pre-CCRT and post-CCRT positive nodal status (both *p* < 0.001), advanced pre-CCRT and post-CCRT tumor status (*p* < 0.001 and *p* = 0.002), perineural invasion (*p* = 0.023), and vascular invasion (*p* < 0.001). In addition, tumors with CTSE overexpression had a remarkably lesser degree of tumor regression (*p* = 0.003). In patients with CTSE overexpression, there were 27 (15.7%) patients with no or little response to CCRT (grade 0–1), 54 (31.4%) patients with a modest response to CCRT (grade 2–3), and 5 (2.9%) patients with a complete response to CCRT (grade 4).

### 3.4. Survival and Prognostic Implications of CTSE Expression in Rectal Carcinoma

**Figure 3:**
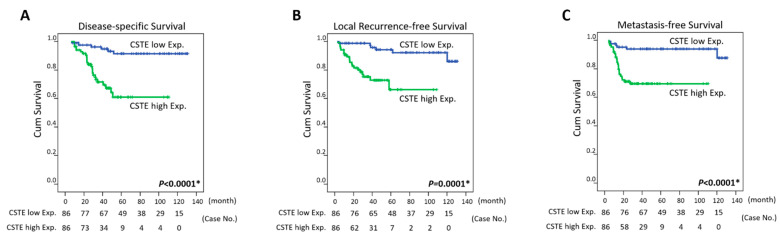
Survival analysis. Almost all patients developed events within 60 months. For the non-eventful patients, the mean follow-up duration was 66.4 months (median 59.2, ranged from 10.3 to 131.3). Kaplan-Meier curves were drawn and revealed that CTSE overexpression was significantly correlated with poor (**A**) disease-specific survival, (**B**) local recurrence-free survival, and (**C**) metastasis-free survival.

At the univariate level, CTSE overexpression in tumor specimens was an unfavorable prognostic factor for all three endpoints: disease-specific survival (DSS), metastasis-free survival (MeFS) (both *p* < 0.0001), and local recurrence-free survival (LRFS) (*p* = 0.0001) (Table 3 and Figure 3). Clinicopathologic variables, including the tumor regression grade, presence of vascular invasion, pretreatment nodal status, and posttreatment tumor status, were significantly correlated with one of the three endpoints at a minimum. At the multivariate level, CTSE overexpression remained an independent prognostic factor for poor DSS, MeFS (both *p* = 0.005), and LRFS (*p* = 0.019) (Table 4). A lower degree of tumor regression was considerably correlated only with inferior MeFS (*p* = 0.043).

### 3.5. CTSE Overexpression may form a Defensive Mucous Barrier against CCRT

A gene co-expression analysis was conducted to predict the biological functions of CTSE in CRC. We assessed the top 200 genes that were positively correlated (Supplementary Table S1) or negatively correlated (Supplementary Table S2) with *CTSE* from The Cancer Genome Atlas (TCGA) database (n = 594). Using the PANTHER classification system, we identified that the top 1 and 2 biological process terms associated with *CTSE* upregulation were maintenance of gastrointestinal epithelium (GO: 0030277, fold enrichment: 29.56) and epithelial structure maintenance (GO: 0010669, fold enrichment: 21.9), respectively (Supplementary Figure S1A), suggesting that CTSE is functionally correlated with the protective role of the intestinal epithelium. Interestingly, we found that mucin 2 (*MUC2*), the main mucin in the intestines, was involved in these two biological processes, and that many other genes co-upregulated with *CTSE* were also involved in the processes of mucin synthesis. These genes comprised SAM pointed domain containing ETS transcription factor (*SPDEF*) (Spearman’s correlation: 0.594) (Supplementary Figure S2A), *MUC2* (Spearman’s correlation: 0.591) (Supplementary Figure S2B), anterior gradient 2 (*AGR2*) (Spearman’s correlation: 0.58) (Supplementary Figure S2C), and endoplasmic reticulum to nucleus signaling 2 (*ERN2*) (Spearman’s correlation: 0.502) (Supplementary Figure S2D). In addition, genes related to mucus secretion and expansion, including *RAB27B* (Spearman’s correlation: 0.599) (Supplementary Figure S3A) and solute carrier family 4 member 4 (*SLC4A4*) (Spearman’s correlation: 0.584) (Supplementary Figure S3B), were also co-upregulated with *CTSE*. Furthermore, in terms of cellular components, we identified the term specific granule lumen (GO: 0035580, fold enrichment: 7.95) as the most significantly associated with *CTSE* upregulation (Supplementary Figure S1B) and identified the transcobalamin 1 (*TCN1*) gene (Spearman’s correlation: 0.521) (Supplementary Figure S3C) as related to this cellular component. As a vitamin B12 binding protein, TCN1 has been suggested to be an unfavorable prognostic factor among rectal cancer patients undergoing CCRT in our previous study [14].

## 4. Discussion

Neoadjuvant CCRT serves as a standardized treatment for locally advanced rectal cancer before surgical resection or for unresectable patients. However, only approximately 20% of patients undergoing preoperative CCRT achieve a pathologic complete response [15]. Moreover, 15–20% of patients receiving preoperative CCRT progress to local recurrence or distant metastasis [16]. This discouraging situation stresses the urgent need for the identification of valuable predictive and prognostic biomarkers. In this study, we provide the first evidence demonstrating that CTSE overexpression is significantly correlated with inferior clinical outcomes and acts as an unfavorable predictive biomarker for rectal cancer patients treated with preoperative CCRT. In addition, it has been reported that *CTSE* is upregulated in CRC tissue samples compared with normal controls [17], further supporting our findings.

Since CTSE overexpression has been linked to development of many types of cancer, we wondered how specific its overexpression is to CRC. Using the Cancer Cell Line Encyclopedia database (https://portals.broadinstitute.org/ccle/page?gene=CTSE) (accessed on 1 July 2021), we found that the mRNA levels of *CTSE* are higher in gastric, bile duct, pancreatic, and colorectal cancer cell lines. Among these cancer types, we further observed that the transcripts of *CTSE* significantly increase in pancreatic, colon, and rectal tumor samples compared with their paired normal tissues by the Gene Expression Profiling Interactive Analysis database (http://gepia.cancer-pku.cn/detail.php?gene=CTSE) (accessed on 1 July 2021). Although abundantly expressed in gastric cancer cell lines, the *CTSE* mRNA levels do not change between gastric tumor samples and paired normal tissues. In addition, it has also been suggested that the *CTSE* transcripts significantly increase (approximately 100-fold) in intestinal adenoma and carcinoma compared to normal epithelium [18], further highlighting the specific role of CTSE in colorectal cancer.

The intestinal epithelium is composed of many cell types that can work cooperatively to construct a physical barrier against luminal noxious stimuli and coordinate immune responses. Among these cell types, goblet cells are specialized for producing and secreting intestinal mucus. Interestingly, many genes co-upregulated with *CTSE* were involved in this process. The transcription factor SPDEF (Supplementary Figure S2A) is important for goblet cell maturation [19]. MUC2 (Supplementary Figure S2B), expressed specifically in goblet cells, is the main mucin in the intestines and forms a tremendous net-like multimeric mucous barrier. AGR2 (Supplementary Figure S2C) and ERN2 (Supplementary Figure S2D) are two goblet cell-specific endoplasmic reticulum proteins that are indicated to be essential for MUC2 biosynthesis [20,21]. The intestinal mucous barrier can protect the gut lumen from bacterial penetration; however, dysregulated mucin synthesis may cause cancer cells to lose permeability, enabling them to resist chemotherapy [22], and create a defensive barrier against cytotoxic T cell infiltration [23]. Nevertheless, whether *CTSE* and its co-upregulated genes mentioned above contribute to aberrant mucin synthesis and CCRT resistance in rectal cancer needs further investigation.

Mucus can be released from goblet cells by vesicle secretion at a basal rate and by compound exocytosis upon stimulation [24]. *RAB27B* (Supplementary Figure S3A), a member of the Rab family, was identified as the seventh gene most significantly positively correlated with *CTSE*, and it has been suggested to be implicated in vesicular fusion and the basal mucin secretory machinery of the mouse stomach [25]. In addition, triggered by endocytosis of pathogenic bacteria, compound exocytosis leads to rapid release of mucus by goblet cells and goblet cell exhaustion and extrusion from the epithelium, which can prevent bacterial intrusion [26]. CTSE itself is located primarily in the endosome (GO: 0005768), suggesting that it may play a key role in this distinct bacterial endocytosis process. Moreover, for mucus net-like expansion upon secretion, the compactly packed mucin polymers stored in the secretory vesicles of goblet cells must be exposed to decreased Ca^2+^ levels and increased pH [27]. Bicarbonate is the physiological and ideal solution for raising pH and precipitating Ca^2+^ [28]. Interestingly, the *SLC4A4* gene (Supplementary Figure S3B), which is involved in bicarbonate secretion and intracellular pH regulation, was also co-upregulated with *CTSE*. However, the correlations among CTSE, RAB27B, and SLC4A4 expression, uncontrolled mucus secretion and expansion, and CCRT resistance in rectal cancer require further investigation.

In view of the fact that CRC is a heterogeneous and molecularly complex disease, four consensus molecular subtypes (CMSs) with distinct characteristics: CMS1 (microsatellite instability, MSI), CMS2 (canonical), CMS3 (metabolic), and CMS4 (mesenchymal), were proposed to guide treatment more precisely [29]. Therefore, we were interested in checking whether CTSE overexpression is specific to one or more subtypes. Based on the results from gene co-expression analysis and annotation, we found that many genes co-upregulated with *CTSE* were involved in the processes of mucin synthesis, secretion, and expansion. As specialized in producing and secreting mucins, the enrichment of goblet cells has been linked to CMS3 subtype assignment in CRC [30], which is characterized by metabolic dysregulation. This may be due to the enhanced metabolic requirement of goblet cells to produce mucins. Goblet cells can also endocytose luminal antigens and transfer these antigens to antigen-presenting cells in the lamina propria, and these routes for antigen transfer are known as goblet cell-associated antigen passages (GAPs) [31]. GAPs have been suggested to maintain Treg cells, encourage macrophages to produce IL-10, and induce tolerogenic phenotypes in dendritic cells, which may induce tolerance to food antigens [32]. CTSE itself plays a role in antigen processing and the presentation of exogenous peptides via MHC class II (GO: 0019886), implying that it can participate in GAPs. In addition, MUC2 overexpression in ovarian cancer has also been connected to M2 macrophage polarization and poor patient survival [33]. Taken together, in terms of immune landscape, CTSE overexpression is more likely to be associated with CRC CMS4 subtype, which features in immunosuppressive cell infiltration (Tregs, M2 macrophages, and myeloid-derived suppressor cells). These observations further highlight the molecular heterogeneity of CRC and may provide more clues for how such pathways can be therapeutically targeted.

## 5. Conclusions

In this study, we provided evidence that overexpression of the intracellular proteinase CTSE is linked to aggressive clinicopathological parameters and functions as an independent prognostic factor for rectal cancer patients undergoing CCRT. Additionally, a greater comprehension of the role of CTSE in rectal cancer and its association with CCRT efficacy could make CTSE a potential predictive indicator.

## Figures and Tables

**Table 1:** Summary of differentially expressed genes associated with digestion (GO: 0007586) in relation to the response to CCRT in rectal carcinoma.

Probe	Comparison log Ratio	Comparison *p*-Value	Gene Symbol	Gene Name	Biological Process	Molecular Function
1555854_at	0.1462	0.0069	*AKR1C1*	aldo-keto reductase family 1; member C1 (dihydrodiol dehydrogenase 1; 20-alpha (3-alpha)-hydroxysteroid dehydrogenase), aldo-keto reductase family 1; member C2 (dihydrodiol dehydrogenase 2; bile acid binding protein; 3-alpha hydroxysteroid dehydrogenase; type III)	bile acid and bile salt transport, bile acid metabolic process, cholesterol absorption, cholesterol homeostasis, digestion, electron transport, lipid metabolic process, prostaglandin metabolic process, protein homooligomerization, steroid metabolic process, xenobiotic metabolic process	20-alpha-hydroxysteroid dehydrogenase activity, 3-alpha-hydroxysteroid dehydrogenase (A-specific) activity, 3-alpha-hydroxysteroid dehydrogenase (B-specific) activity, aldo-keto reductase activity, bile acid binding, carboxylic acid binding, oxidoreductase activity, trans-1;2-dihydrobenzene-1;2-diol dehydrogenase activity
204151_x_at	0.7096	0.003	*AKR1C1*	aldo-keto reductase family 1; member C1 (dihydrodiol dehydrogenase 1; 20-alpha (3-alpha)-hydroxysteroid dehydrogenase), aldo-keto reductase family 1; member C2 (dihydrodiol dehydrogenase 2; bile acid binding protein; 3-alpha hydroxysteroid dehydrogenase; type III)	bile acid and bile salt transport, bile acid metabolic process, cholesterol absorption, cholesterol homeostasis, digestion, electron transport, lipid metabolic process, prostaglandin metabolic process, protein homooligomerization, steroid metabolic process, xenobiotic metabolic process	20-alpha-hydroxysteroid dehydrogenase activity, 3-alpha-hydroxysteroid dehydrogenase (A-specific) activity, 3-alpha-hydroxysteroid dehydrogenase (B-specific) activity, aldo-keto reductase activity, bile acid binding, carboxylic acid binding, oxidoreductase activity, trans-1;2-dihydrobenzene-1;2-diol dehydrogenase activity
205009_at	0.9983	0.0022	*TFF1*	trefoil factor 1	carbohydrate metabolic process, cell differentiation, defense response, digestion, negative regulation of cell proliferation	20-alpha-hydroxysteroid dehydrogenase activity, 3-alpha-hydroxysteroid dehydrogenase (A-specific) activity, 3-alpha-hydroxysteroid dehydrogenase (B-specific) activity, aldo-keto reductase activity, bile acid binding, carboxylic acid binding, oxidoreductase activity, trans-1;2-dihydrobenzene-1;2-diol dehydrogenase activity
205869_at	0.1705	0.0076	*PRSS1*	protease; serine; 1 (trypsin 1)	digestion, proteolysis	growth factor activity, protein binding
205927_s_at	1.7848	<0.0001	*CTSE*	cathepsin E	antigen processing and presentation of exogenous peptide antigen via MHC class II, digestion, proteolysis	calcium ion binding, hydrolase activity, metal ion binding, peptidase activity, serine-type endopeptidase activity, trypsin activity
206023_at	0.8363	0.0076	*NMU*	neuromedin U	G-protein coupled receptor protein signaling pathway, digestion, neuropeptide signaling pathway, regulation of smooth muscle contraction, signal transduction	aspartic-type endopeptidase activity, cathepsin E activity, hydrolase activity, pepsin A activity, peptidase activity
207080_s_at	0.7389	0.0092	*PYY*	peptide YY	G-protein coupled receptor protein signaling pathway, cell motility, cell proliferation, cell-cell signaling, cytoskeleton organization and biogenesis, digestion, feeding behavior	receptor binding
208596_s_at	0.6909	0.0091	*UGT1A1*	UDP glucuronosyltransferase 1 family; polypeptide A1, UDP glucuronosyltransferase 1 family; polypeptide A10, UDP glucuronosyltransferase 1 family; polypeptide A3, UDP glucuronosyltransferase 1 family; polypeptide A4, UDP glucuronosyltransferase 1 family; polypeptide A5, UDP glucuronosyltransferase 1 family; polypeptide A6, UDP glucuronosyltransferase 1 family; polypeptide A7, UDP glucuronosyltransferase 1 family; polypeptide A8, UDP glucuronosyltransferase 1 family; polypeptide A9	bilirubin conjugation, digestion, estrogen metabolic process, metabolic process, xenobiotic metabolic process	hormone activity
213921_at	0.8083	0.002	*SST*	somatostatin	G-protein coupled receptor protein signaling pathway, cell surface receptor linked signal transduction, cell-cell signaling, digestion, induction of apoptosis by hormones, negative regulation of cell proliferation, regulation of cell migration, response to nutrient, synaptic transmission	UDP-glycosyltransferase activity, glucuronosyltransferase activity, transferase activity, transferase activity; transferring glycosyl groups, transferase activity; transferring hexosyl groups
214476_at	0.4628	0.0024	*TFF2*	trefoil factor 2 (spasmolytic protein 1)	defense response, digestion	hormone activity
215125_s_at	0.8769	0.0065	*UGT1A1*	UDP glucuronosyltransferase 1 family; polypeptide A1, UDP glucuronosyltransferase 1 family; polypeptide A10, UDP glucuronosyltransferase 1 family; polypeptide A3, UDP glucuronosyltransferase 1 family; polypeptide A4, UDP glucuronosyltransferase 1 family; polypeptide A5, UDP glucuronosyltransferase 1 family; polypeptide A6, UDP glucuronosyltransferase 1 family; polypeptide A7, UDP glucuronosyltransferase 1 family; polypeptide A8, UDP glucuronosyltransferase 1 family; polypeptide A9	bilirubin conjugation, digestion, estrogen metabolic process, metabolic process, xenobiotic metabolic process	
221305_s_at	0.4507	0.0039	*UGT1A8*	UDP glucuronosyltransferase 1 family; polypeptide A8, UDP glucuronosyltransferase 1 family; polypeptide A9	bilirubin conjugation, digestion, estrogen metabolic process, metabolic process, xenobiotic metabolic process	UDP-glycosyltransferase activity, glucuronosyltransferase activity, transferase activity, transferase activity; transferring glycosyl groups, transferase activity; transferring hexosyl groups
206293_at	−0.3299	0.0003	*SULT2A1*	Sulfotransferase family; cytosolic; 2A; dehydroepiandrosterone (DHEA)-preferring; member 1	bile acid catabolic process, digestion, lipid metabolic process, steroid metabolic process	bile-salt sulfotransferase activity, sulfotransferase activity, transferase activity

**Table 2:** Correlations between CTSE expression and clinicopathological factors in 172 rectal cancer patients receiving neoadjuvant CCRT.

Parameter		No.	CTSE Expression		*p*-Value
			Low Exp.	High Exp.	
Gender	Male	108	57	51	0.334
	Female	64	29	35	
Age	<70	106	56	50	0.347
	≥70	66	30	36	
Pre-Tx tumor status (Pre-T)	T1-T2	81	54	27	<0.001 *
	T3-T4	91	32	59	
Pre-Tx nodal status (Pre-N)	N0	125	76	49	<0.001 *
	N1-N2	47	10	37	
Post-Tx tumor status (Post-T)	T1-T2	86	53	33	0.002 *
	T3-T4	86	33	53	
Post-Tx nodal status (Post-N)	N0	123	72	51	<0.001 *
	N1-N2	49	14	35	
Vascular invasion	Absent	157	85	72	<0.001 *
	Present	15	1	14	
Perineural invasion	Absent	167	86	81	0.023 *
	Present	5	0	5	
Tumor regression grade	Grade 0-1	37	10	27	0.003 *
	Grade 2~3	118	64	54	
	Grade 4	17	12	5	

Tx, treatment; * statistically significant.

**Table 3:** Univariate log-rank analysis for important clinicopathological variables and CTSE expression.

Parameter		No. of Case	DSS		LRFS		MeFS			
			No. of Event	*p*-Value	No. of Event	*p*-Value	No. of Event		*p*-Value	
Gender	Male	108	20	0.9026	7	0.2250	17		0.3520	
	Female	64	11		20		14			
Age	<70	106	19	0.8540	18	0.6615	20		0.7427	
	≥70	66	12		9		11			
Pre-Tx tumor status (Pre-T)	T1-T2	81	10	0.0776	10	0.2261	11		0.1745	
	T3-T4	91	21		17		20			
Pre-Tx nodal status (Pre-N)	N0	125	19	0.0711	15	0.0070 *	19		0.0973	
	N1-N2	47	21		12		12			
Post-Tx tumor status (Post-T)	T1-T2	86	7	0.0006 *	7	0.0040 *	8		0.0033 *	
	T3-T4	86	24		20		23			
Post-Tx nodal status (Post-N)	N0	123	21	0.5998	16	0.1320	20		0.4634	
	N1-N2	49	10		11		11			
Vascular invasion	Absent	157	25	0.0184*	21	0.0028 *	27		0.4470	
	Present	15	6		6		4			
Perineural invasion	Absent	167	29	0.2559	25	0.0940	30		0.9083	
	Present	5	2		2		1			
Tumor regression grade	Grade 0-1	37	13	0.0038 *	10	0.0090*	14		0.0006 *	
	Grade 2~3	118	17		17		16			
	Grade 4	17	1		0		1			
Down stage after CCRT	Non-Sig.	150	29	0.1651	24	0.5961	30		0.0853	
	Sig. (≥2)	22	2		3		1			
CTSE expression	Low Exp.	86	6	<0.0001 *	6	0.0001*	6		<0.0001 *
	High Exp.	86	25		25		21			

DSS, disease-specific survival; LRFS, local recurrence-free survival; MeFS, metastasis-free survival; * statistically significant.

**Table 4:** Multivariate analysis.

Parameter	DSS			LRFS			MeFS		
	H.R	95% CI	*p*-Value	H.R	95% CI	*p*-Value	H.R	95% CI	*p*-Value
Tumor regression grade	1.754	0.867–3.546	0.118	2.132	0.994–9.132	0.052	2.049	1.022–4.115	0.043 *
CTSE expression	3.901	1.514–10.046	0.005 *	3.748	1.243–11.306	0.019 *	4.123	1.538–11.059	0.005 *
Vascular invasion	1.701	0.660–4.384	0.272	2.083	0.763–5.682	0.152	-	-	-
Post-Tx tumor status (Post-T)	2.335	0.955–5.706	0.063	1.806	0.727–4.489	0.203	1.827	0.781–4.271	0.164
Pre-Tx nodal status (Pre-N)	-	-	-	1.358	0.570–3.237	0.489	-	-	-

DSS, disease-specific survival; LRFS, local recurrence-free survival; MeFS, metastasis-free survival; * statistically significant.

## Data Availability

The dataset analyzed in the current study (GSE35452) is available in a public transcriptome dataset from the Gene Expression Omnibus (GEO) database (National Center for Biotechnology Information, Bethesda, MD, USA).

## Supplementary Materials

The following are available online at https://www.mdpi.com/article/10.3390/life11070646/s1, Figure S1: The biological process and cellular component terms enriched with CTSE upregulation, Figure S2. Associations among *CTSE,* SPDEF, MUC2, AGR2, and ERN2 gene expression, Figure S3. Associations among *CTSE,* RAB27B, SLC4A4, and TCN1 gene expression, Table S1. The top 200 genes positively correlated with *CTSE*, Table S2. The top 200 genes negatively correlated with *CTSE*.
