# Supplementary material for: CTSE Overexpression Is an Adverse Prognostic Factor for Survival among Rectal Cancer Patients Receiving CCRT
Source: Life (Basel). 2021 Jul 2;11(7):646. doi: 10.3390/life11070646 (PMC8304221; doi:10.3390/life11070646)
Supplement: Supplementary file 1 [file life-11-00646-s001.zip › life-1255863-supplementary.pdf]

life

MDPI

Article

CTSE Overexpression is an Adverse Prognostic Factor for Survival among Rectal Cancer Patients Receiving CCRT

Chia-Lin Chou [1], Tzu-Ju Chen [2,3,4], Yu-Feng Tian [1], Ti-Chun Chan [5,6], Cheng-Fa Yeh [7], Wan-Shan Li [3,8], Hsin-Hwa Tsai [2,5], Chien-Feng Li [2,5,6,8,9,*], Hong-Yue Lai [2,5,*]

[1] Division of Colon and Rectal Surgery, Department of Surgery, Chi Mei Medical Center, Tainan 710, Taiwan; 991101@mail.chimei.org.tw (C.-L.C.); d870722@mail.chimei.org.tw (Y.-F.T.);
[2] Department of Clinical Pathology, Chi Mei Medical Center, Tainan 710, Taiwan; a108n2@mail.chimei.org.tw (T.-J.C.); livelychord.tsai@biocheck.com.tw (H.-H.T.)
[3] Department of Medical Technology, Chung Hwa University of Medical Technology, Tainan 717, Taiwan; a80818@mail.chimei.org.tw
[4] Institute of Biomedical Sciences, National Sun Yat-Sen University, Kaohsiung 804, Taiwan
[5] Department of Medical Research, Chi Mei Medical Center, Tainan 710, Taiwan; 090807@nhri.edu.tw
[6] National Institute of Cancer Research, National Health Research Institutes, Tainan 704, Taiwan
[7] Department of Internal Medicine, Chi Mei Medical Center, Tainan 710, Taiwan; 970402@mail.chimei.org.tw
[8] Institute of Precision Medicine, National Sun Yat-Sen University, Kaohsiung 804, Taiwan
[9] Department of Pathology, School of Medicine, College of Medicine, Kaohsiung Medical University, Kaohsiung 807, Taiwan
* Correspondence: cfli@mail.chimei.org.tw (C.-F.L.); b00137@mail.chimei.org.tw (H.-Y.L.)

Abstract: The introduction of preoperative concurrent chemoradiotherapy (CCRT) increases the rate of anal preservation and allows tumor downstaging for clinical stage T3/T4 or node-positive rectal cancer patients. However, there is no precise predictive tool to verify the presence of residual tumor apart from surgical resection. The gastrointestinal (GI) tract not only digests nutrients but also coordinates immune responses. As the outermost layer of the GI tract, mucus plays a key role in mediating the interaction between the digestive and immune systems, and aberrant mucus mesh formation may cause chemoresistance by impeding drug delivery. However, the correlations among digestion-related genes, mucin synthesis, and chemoresistance remain poorly understood. In the present study, we evaluated genes related to digestion (GO: 0007586) and identified cathepsin E (*CTSE*), which is involved in immune regulation, as the most significantly upregulated gene associated with CCRT resistance in rectal cancer in a public transcriptome dataset (GSE35452). We recovered 172 records of rectal cancer patients receiving CCRT followed by surgical resection from our biobank and evaluated the expression level of CTSE using immunohistochemistry. The results revealed that tumors with CTSE overexpression were significantly correlated with pre-CCRT and post-CCRT positive nodal status (both $p < 0.001$), advanced pre-CCRT and post-CCRT tumor status ($p < 0.001$ and $p = 0.002$), perineural invasion ($p = 0.023$), vascular invasion ($p < 0.001$), and a lesser degree of tumor regression ($p = 0.003$). At the univariate level, CTSE overexpression was an adverse prognostic factor for all three endpoints: disease-specific survival (DSS), metastasis-free survival (MeFS) (both $p < 0.0001$), and local recurrence-free survival (LRFS) ($p = 0.0001$). At the multivariate level, CTSE overexpression remained an independent prognostic factor for poor DSS, MeFS (both $p = 0.005$), and LRFS ($p = 0.019$). Through bioinformatics analysis, we speculated that CTSE overexpression may confer CCRT resistance by forming a defensive mucous barrier. Taken together, these results suggest that CTSE overexpression is related to CCRT resistance and inferior survival in rectal cancer patients, highlighting the potential predictive and prognostic value of CTSE expression.

Keywords: rectal cancer; chemoradiotherapy; CTSE; digestion; immune response

Citation: Chou, C.-L.; Chen, T.-J.; Tian, Y.-F.; Chan, T.-C.; Yeh, C.-F.; Li, W.-S.; Tsai, H.-H.; Li, C.-F.; Lai, H.-Y. CTSE Overexpression is an Adverse Prognostic Factor for Survival among Rectal Cancer Patients Receiving CCRT. *Life* **2021**, *11*, 646. https://doi.org/10.3390/life11070646

Academic Editor: Mattia Cappelletti and Michela Carola Speciani

Received: 26 May 2021
Accepted: 29 June 2021
Published: 2 July 2021

Publisher's Note: MDPI stays neutral with regard to jurisdictional claims in published maps and institutional affiliations.

Copyright: © 2021 by the authors. Submitted for possible open access publication under the terms and conditions of the Creative Commons Attribution (CC BY) license (http://creativecommons.org/licenses/by/4.0/).

Supplementary Materials:

Table S1. The top 200 ***genes positively correlated with CTSE.***

Correlated Gene	Cytoband	Spearman's Correlation	p-Value	q-Value
**REG4**	1p12	0.759	6.01E-112	**1.20E-107**
**MMP28**	17q12	0.629	1.84E-66	**1.84E-62**
**GCNT3**	15q22.2	0.628	3.19E-66	**2.13E-62**
**FAM177B**	1q41	0.622	9.76E-65	**4.88E-61**
**PLLP**	16q13	0.615	7.06E-63	**2.67E-59**
**TRIM7**	5q35.3	0.615	8.01E-63	**2.67E-59**
**RAB27B**	18q21.2	0.599	6.52E-59	**1.86E-55**
**SPDEF**	6p21.31	0.594	7.92E-58	**1.98E-54**
**ZBTB7C**	18q21.1	0.593	1.66E-57	**3.68E-54**
**MUC2**	11p15.5	0.591	5.71E-57	**1.04E-53**
**ADGRF1**	6p12.3|6	0.587	4.02E-56	**6.71E-53**
**SLC4A4**	4q13.3	0.584	1.64E-55	**2.52E-52**
**TSPAN1**	1p34.1	0.584	2.15E-55	**3.08E-52**
**AGR2**	7p21.1	0.58	1.45E-54	**1.94E-51**
**BARX2**	11q24.3	0.575	1.97E-53	**2.47E-50**
**B3GALT5**	21q22.2	0.574	3.42E-53	**4.03E-50**
**SPINK4**	9p13.3	0.571	1.48E-52	**1.65E-49**
**CREB3L1**	11p11.2	0.569	5.32E-52	**5.61E-49**
**HSD17B2**	16q23.3	0.568	9.12E-52	**9.13E-49**
**FER1L6**	8q24.13	0.566	1.64E-51	**1.56E-48**
**SDR16C5**	8q12.1	0.565	3.18E-51	**2.76E-48**
**TENT5A**	6q14.1	0.564	5.67E-51	**4.73E-48**
**CCDC68**	18q21.2	0.562	1.55E-50	**1.24E-47**
**PLA2G2A**	1p36.13	0.559	6.63E-50	**5.10E-47**
**FFAR4**	10q23.33	0.558	9.72E-50	**6.95E-47**
**DUSP4**	8p12	0.557	1.60E-49	**1.07E-46**
**VSTM5**	11q21	0.556	2.51E-49	**1.62E-46**
**VNN1**	6q23.2	0.555	5.01E-49	**3.14E-46**
**MYRF**	11q12.2	0.553	9.31E-49	**5.65E-46**
**CA2**	8q21.2	0.553	1.09E-48	**6.44E-46**
**TFF2**	21q22.3	0.552	1.77E-48	**1.01E-45**
**CHST5**	16q23.1	0.551	2.11E-48	**1.17E-45**
**CD55**	1q32.2	0.549	6.13E-48	**3.23E-45**
**RNF125**	18q12.1	0.547	1.83E-47	**9.39E-45**
**QSOX1**	1q25.2	0.547	1.91E-47	**9.54E-45**
**ABHD3**	18q11.2	0.546	3.20E-47	**1.49E-44**
**VSIG2**	11q24.2	0.545	3.63E-47	**1.65E-44**
**TFF1**	21q22.3	0.539	5.38E-46	**2.29E-43**
**FCGBP**	19q13.2	0.536	2.54E-45	**1.04E-42**
**AGR3**	7p21.1	0.534	5.58E-45	**2.19E-42**
**RIOK3**	18q11.2	0.533	1.11E-44	**4.28E-42**
**F3**	1p21.3	0.531	1.87E-44	**6.93E-42**
**MLPH**	2q37.3	0.531	1.87E-44	**6.93E-42**
**PPP4R1**	18p11.22	0.531	2.44E-44	**8.87E-42**
**KCTD1**	18q11.2	0.529	5.05E-44	**1.79E-41**
**S100A16**	1q21.3	0.529	5.09E-44	**1.79E-41**
**TCN1**	11q12.1	0.521	1.73E-42	**5.76E-40**
**C2ORF88**	2q32.2	0.518	4.89E-42	**1.58E-39**
**AFAP1L2**	10q25.3	0.513	4.29E-41	**1.30E-38**
**CANT1**	17q25.3	0.512	7.34E-41	**2.19E-38**
**MCU**	10q22.1	0.511	1.27E-40	**3.70E-38**
**VAPA**	18p11.22	0.51	1.39E-40	**3.99E-38**
**PLAC8**	4q21.22	0.509	2.28E-40	**6.26E-38**
**KLK11**	19q13.41	0.508	3.17E-40	**8.47E-38**
**KIAA1211**	4q12	0.506	8.13E-40	**2.14E-37**

**DHRS9**	2q31.1	0.504	1.58E-39	**4.10E-37**
**KLF4**	9q31.2	0.504	1.70E-39	**4.36E-37**
**LIMA1**	12q13.12	0.504	1.89E-39	**4.78E-37**
**IL1R2**	2q11.2	0.504	1.93E-39	**4.83E-37**
**ALDH1L1**	3q21.3	0.503	3.34E-39	**8.25E-37**
**ERN2**	16p12.2	0.502	4.74E-39	**1.13E-36**
**KCNK1**	1q42.2	0.499	1.14E-38	**2.56E-36**
**SERPINB5**	18q21.33	0.497	2.96E-38	**6.38E-36**
**IL1RN**	2q14.1	0.497	3.54E-38	**7.53E-36**
**CHP1**	15q15.1	0.496	4.76E-38	**1.00E-35**
**EGLN3**	14q13.1	0.495	7.43E-38	**1.53E-35**
**GJB5**	1p34.3	0.493	1.57E-37	**3.11E-35**
**HYAL1**	3p21.31	0.492	2.02E-37	**3.92E-35**
**PTGER2**	14q22.1	0.491	3.56E-37	**6.75E-35**
**LYZ**	12q15	0.489	5.55E-37	**1.04E-34**
**LIPH**	3q27.2	0.488	9.64E-37	**1.77E-34**
**SERPINB1**	6p25.2	0.487	1.50E-36	**2.72E-34**
**ITLN1**	1q23.3	0.486	2.34E-36	**4.10E-34**
**SERPINB6**	6p25.2	0.484	3.87E-36	**6.74E-34**
**GSKIP**	14q32.2	0.484	5.01E-36	**8.57E-34**
**ANXA10**	4q32.3	0.592	6.22E-36	**1.05E-33**
**VPS37B**	12q24.31	0.482	9.43E-36	**1.55E-33**
**B3GNT7**	2q37.1|2q37.1	0.481	1.45E-35	**2.30E-33**
**B3GNT6**	11q13.5	0.48	1.65E-35	**2.60E-33**
**ST3GAL4**	11q24.2	0.48	1.80E-35	**2.81E-33**
**CIDEC**	3p25.3	0.48	2.07E-35	**3.22E-33**
**FAM189A2**	9q21.12	0.479	2.50E-35	**3.84E-33**
**AQP3**	9p13.3	0.479	2.67E-35	**4.08E-33**
**INPP1**	2q32.2	0.478	3.54E-35	**5.34E-33**
**SEMA4B**	15q26.1	0.478	3.55E-35	**5.34E-33**
**TMEM61**	1p32.3	0.478	3.82E-35	**5.70E-33**
**VSIG1**	Xq22.3	0.477	4.90E-35	**7.16E-33**
**ST6GALNAC6**	9q34.11	0.476	9.13E-35	**1.31E-32**
**VILL**	3p22.2	0.475	1.01E-34	**1.44E-32**
**SERPINB8**	18q22.1	0.475	1.09E-34	**1.52E-32**
**TRIM40**	6p22.1	0.474	1.53E-34	**2.07E-32**
**VWA3B**	2q11.2	0.473	2.64E-34	**3.46E-32**
**C4BPB**	1q32.1	0.472	3.71E-34	**4.79E-32**
**CRIP1**	14q32.33	0.47	8.28E-34	**1.06E-31**
**ASPHD2**	22q12.1	0.469	1.08E-33	**1.37E-31**
**NAPG**	18p11.22	0.468	1.32E-33	**1.66E-31**
**TMEM92**	17q21.33	0.468	1.43E-33	**1.76E-31**
**CHST6**	16q23.1	0.468	1.46E-33	**1.80E-31**
**CLDN18**	3q22.3	0.468	1.67E-33	**2.03E-31**
**GABRP**	5q35.1	0.467	2.29E-33	**2.71E-31**
**SLC37A1**	21q22.3	0.467	2.34E-33	**2.76E-31**
**SOCS6**	18q22.2	0.466	2.58E-33	**3.03E-31**
**PDE4D**	5q11.2-q12.1	0.466	2.76E-33	**3.21E-31**
**GFI1**	1p22.1	0.465	3.62E-33	**4.17E-31**
**ADGRG6**	6q24.2	0.464	5.32E-33	**6.09E-31**
**HPSE**	4q21.23	0.464	5.42E-33	**6.17E-31**
**CYP2C18**	10q23.33	0.463	7.42E-33	**8.35E-31**
**DAPK1**	9q21.33	0.463	9.18E-33	**1.03E-30**
**TOX**	8q12.1	0.463	1.02E-32	**1.14E-30**
**SRD5A3**	4q12	0.462	1.21E-32	**1.33E-30**
**FAM114A1**	4p14	0.461	1.63E-32	**1.78E-30**
**MAP3K6**	1p36.11	0.461	1.71E-32	**1.86E-30**
**SDCBP2**	20p13	0.461	1.85E-32	**2.00E-30**
**CYSTM1**	5q31.3	0.461	1.98E-32	**2.12E-30**
**TRIM16**	17p12	0.46	2.30E-32	**2.43E-30**

**SHROOM3**	4q21.1	0.46	2.49E-32	**2.62E-30**
**RHBDL2**	1p34.3	0.459	3.49E-32	**3.66E-30**
**ANXA2P2**	9p13.3	0.458	5.12E-32	**5.29E-30**
**LRRC26**	9q34.3	0.457	7.14E-32	**7.22E-30**
**SEMG1**	20q13.12	0.563	7.41E-32	**7.46E-30**
**SLC1A1**	9p24.2	0.456	8.59E-32	**8.59E-30**
**STYK1**	12p13.2	0.456	9.48E-32	**9.45E-30**
**ATP2A3**	17p13.2	0.456	1.01E-31	**1.00E-29**
**S100A14**	1q21.3	0.456	1.15E-31	**1.13E-29**
**TRPV6**	7q34	0.455	1.32E-31	**1.29E-29**
**TC2N**	14q32.12	0.454	1.72E-31	**1.66E-29**
**NXF3**	Xq22.1	0.454	2.01E-31	**1.92E-29**
**PTPRH**	19q13.42	0.451	4.59E-31	**4.26E-29**
**DGKA**	12q13.2	0.45	6.86E-31	**6.33E-29**
**SLC6A14**	Xq23	0.45	7.16E-31	**6.58E-29**
**MUC4**	3q29	0.449	1.00E-30	**9.18E-29**
**STIM2**	4p15.2	0.449	1.18E-30	**1.07E-28**
**IQGAP2**	5q13.3	0.449	1.21E-30	**1.09E-28**
**MUC1**	1q22	0.448	1.27E-30	**1.14E-28**
**MYL12B**	18p11.31	0.448	1.57E-30	**1.40E-28**
**TNFSF13**	17p13.1	0.448	1.59E-30	**1.41E-28**
**SPTSSB**	3q26.1	0.447	1.78E-30	**1.56E-28**
**ADTRP**	6p24.1	0.446	2.88E-30	**2.49E-28**
**C1ORF21**	1q25.3	0.446	3.24E-30	**2.80E-28**
**TNFRSF11A**	18q21.33	0.445	3.42E-30	**2.93E-28**
**SIAE**	11q24.2	0.445	3.49E-30	**2.98E-28**
**FRMD3**	9q21.32	0.445	4.55E-30	**3.86E-28**
**RHOF**	12q24.31	0.444	5.24E-30	**4.40E-28**
**SGMS1**	10q11.23	0.444	6.12E-30	**5.06E-28**
**RILP**	17p13.3	0.443	7.79E-30	**6.41E-28**
**ITGB7**	12q13.13	0.443	7.81E-30	**6.41E-28**
**LPCAT4**	15q14	0.442	1.05E-29	**8.56E-28**
**RALBP1**	18p11.22	0.442	1.07E-29	**8.64E-28**
**ANXA2**	15q22.2	0.441	1.33E-29	**1.07E-27**
**ST6GALNAC1**	17q25.1	0.44	1.77E-29	**1.41E-27**
**STS**	Xp22.31	0.44	2.03E-29	**1.61E-27**
**C2ORF72**	2q37.1	0.44	2.15E-29	**1.68E-27**
**SLC17A5**	6q13	0.44	2.19E-29	**1.71E-27**
**BCAS1**	20q13.2	0.438	3.39E-29	**2.58E-27**
**RAP1GAP**	1p36.12	0.437	4.90E-29	**3.72E-27**
**PLA2G4A**	1q31.1	0.437	5.69E-29	**4.27E-27**
**MPDU1**	17p13.1	0.437	6.18E-29	**4.60E-27**
**BACE2**	21q22.2-q22.3	0.436	7.64E-29	**5.64E-27**
**SGPP2**	2q36.1	0.436	8.52E-29	**6.27E-27**
**SLC41A2**	12q23.3	0.434	1.27E-28	**9.17E-27**
**HID1**	17q25.1	0.432	2.32E-28	**1.65E-26**
**GAREM1**	18q12.1	0.432	2.36E-28	**1.67E-26**
**CLCA4**	1p22.3	0.43	4.57E-28	**3.21E-26**
**REP15**	12p11.22	0.43	5.42E-28	**3.79E-26**
**MAP3K5**	6q23.3	0.429	7.19E-28	**5.02E-26**
**SGMS2**	4q25	0.429	7.63E-28	**5.28E-26**
**MYL12A**	18p11.31	0.427	1.07E-27	**7.35E-26**
**RAB26**	16p13.3	0.427	1.24E-27	**8.47E-26**
**SI**	3q26.1	0.528	1.41E-27	**9.53E-26**
**CHRNA7**	15q13.3	0.426	1.45E-27	**9.79E-26**
**HS3ST1**	4p15.33	0.425	2.14E-27	**1.43E-25**
**C4ORF19**	4p14	0.425	2.45E-27	**1.63E-25**
**LRP10**	14q11.2	0.424	2.94E-27	**1.94E-25**
**KDELR3**	22q13.1	0.424	3.24E-27	**2.12E-25**
**FUT8**	14q23.3	0.424	3.63E-27	**2.36E-25**

CDC42EP1	22q13.1	0.422	5.30E-27	**3.42E-25**
CATSPERB	14q32.12	0.422	6.04E-27	**3.89E-25**
RELL1	4p14	0.422	6.30E-27	**4.04E-25**
ARX	Xp21.3	0.421	7.29E-27	**4.65E-25**
SIDT1	3q13.2	0.421	7.61E-27	**4.83E-25**
RARRES1	3q25.32	0.42	9.34E-27	**5.88E-25**
ETHE1	19q13.31	0.42	1.24E-26	**7.75E-25**
ANG	14q11.2	0.419	1.30E-26	**8.11E-25**
ABCA12	2q35	0.419	1.47E-26	**9.19E-25**
TRIB2	2p24.3	0.417	2.28E-26	**1.41E-24**
NRAP	10q25.3	0.417	2.53E-26	**1.56E-24**
TRIM16L	17p11.2	0.417	2.53E-26	**1.56E-24**
SEC24D	4q26	0.417	2.55E-26	**1.56E-24**
NDEL1	17p13.1	0.417	2.58E-26	**1.58E-24**
CHRFAM7A	15q13.2	0.417	2.82E-26	**1.71E-24**
PITPNM3	17p13.2-p13.1	0.417	2.82E-26	**1.71E-24**
HEPACAM2	7q21.2	0.415	4.17E-26	**2.51E-24**
TRNP1	1p36.11	0.415	4.70E-26	**2.82E-24**
ATP9B	18q23	0.415	4.97E-26	**2.97E-24**
TM4SF4	3q25.1	0.414	5.67E-26	**3.38E-24**
SLITRK6	13q31.1	0.414	6.21E-26	**3.69E-24**
TNFAIP8	5q23.1	0.414	7.03E-26	**4.14E-24**
XKR9	8q13.3	0.413	8.69E-26	**5.06E-24**
FAM83A	8q24.13	0.413	9.84E-26	**5.67E-24**
NDUFV2	18p11.22	0.412	1.05E-25	**6.02E-24**

Table S2. The top 200 ***genes negatively correlated with CTSE.***

Correlated Gene	Cytoband	Spearman's Correlation	p-Value	q-Value
POFUT1	20q11.21	-0.593	2.12E-57	**4.25E-54**
CDK5RAP1	20q11.21	-0.566	1.78E-51	**1.62E-48**
SLC5A6	2p23.3	-0.558	8.78E-50	**6.51E-47**
DDX27	20q13.13	-0.557	1.61E-49	**1.07E-46**
PIGU	20q11.22	-0.551	2.47E-48	**1.34E-45**
ASXL1	20q11.21	-0.546	2.20E-47	**1.07E-44**
TTI1	20q11.23	-0.546	2.88E-47	**1.37E-44**
NFS1	20q11.22	-0.544	6.49E-47	**2.89E-44**
DHX35	20q11.23-q12	-0.541	2.12E-46	**9.23E-44**
PLAGL2	20q11.21	-0.536	2.19E-45	**9.15E-43**
AAR2	20q11.23	-0.536	2.70E-45	**1.08E-42**
NELFCD	20q13.32	-0.527	1.14E-43	**3.93E-41**
EIF2S2	20q11.22	-0.526	2.22E-43	**7.54E-41**
CTNNBL1	20q11.23	-0.521	1.90E-42	**6.23E-40**
ZSWIM3	20q13.12	-0.518	7.11E-42	**2.26E-39**
ADNP	20q13.13	-0.515	1.71E-41	**5.35E-39**
PHF20	20q11.22-q11.23	-0.514	2.96E-41	**9.11E-39**
GNG4	1q42.3	-0.511	9.17E-41	**2.70E-38**
TM9SF4	20q11.21	-0.51	1.44E-40	**4.07E-38**
RTF2	20q13.31	-0.509	2.00E-40	**5.57E-38**
LY6G6D	6p21.33	-0.509	2.34E-40	**6.34E-38**
LSM14B	20q13.33	-0.502	3.98E-39	**9.63E-37**
SHROOM4	Xp11.22	-0.502	3.99E-39	**9.63E-37**
TAF4	20q13.33	-0.501	6.26E-39	**1.47E-36**
STX16	20q13.32	-0.501	7.05E-39	**1.64E-36**
ACTR5	20q11.23	-0.5	7.97E-39	**1.83E-36**
TNNC2	20q13.12	-0.5	1.05E-38	**2.40E-36**
RPIA	2p11.2	-0.499	1.40E-38	**3.12E-36**
GID8	20q13.33	-0.498	1.85E-38	**4.07E-36**
CEP250	20q11.22	-0.498	2.02E-38	**4.39E-36**
RNF114	20q13.13	-0.495	6.00E-38	**1.25E-35**

**ELMO2**	20q13.12	-0.494	9.43E-38	**1.93E-35**
**PFDN4**	20q13.2	-0.494	1.10E-37	**2.22E-35**
**TOMM34**	20q13.12	-0.493	1.49E-37	**2.99E-35**
**STAU1**	20q13.13	-0.492	1.85E-37	**3.64E-35**
**PRPF6**	20q13.33	-0.491	3.13E-37	**6.02E-35**
**C11ORF95**	11q13.1	-0.491	3.58E-37	**6.75E-35**
**YTHDF1**	20q13.33	-0.489	7.42E-37	**1.38E-34**
**RBM39**	20q11.22	-0.486	1.73E-36	**3.11E-34**
**UQCC1**	20q11.22	-0.486	1.86E-36	**3.33E-34**
**ANKRD27**	19q13.11	-0.486	2.19E-36	**3.88E-34**
**RAE1**	20q13.31	-0.484	4.99E-36	**8.57E-34**
**NCOA6**	20q11.22	-0.483	5.99E-36	**1.02E-33**
**TRPC4AP**	20q11.22	-0.483	7.51E-36	**1.25E-33**
**CPNE1**	20q11.22	-0.482	8.48E-36	**1.40E-33**
**BCL11A**	2p16.1	-0.481	1.18E-35	**1.91E-33**
**RPRD1B**	20q11.23	-0.481	1.21E-35	**1.96E-33**
**PPP1R3D**	20q13.33	-0.481	1.25E-35	**2.00E-33**
**CEL**	9q34.13	-0.478	4.25E-35	**6.31E-33**
**MAPRE1**	20q11.21	-0.478	4.45E-35	**6.55E-33**
**SLC19A3**	2q36.3	-0.477	5.99E-35	**8.69E-33**
**NORAD**	20q11.23	-0.476	7.54E-35	**1.09E-32**
**CTSV**	9q22.33	-0.475	1.05E-34	**1.47E-32**
**TCFL5**	20q13.33	-0.475	1.16E-34	**1.61E-32**
**DPM1**	20q13.13	-0.475	1.17E-34	**1.61E-32**
**DYNLRB1**	20q11.22	-0.474	1.49E-34	**2.04E-32**
**TP53RK**	20q13.12	-0.474	1.51E-34	**2.06E-32**
**R3HDML**	20q13.12	-0.474	1.66E-34	**2.23E-32**
**MOCS3**	20q13.13	-0.474	1.71E-34	**2.28E-32**
**VAPB**	20q13.32	-0.474	1.79E-34	**2.37E-32**
**SNHG11**	20q11.23	-0.473	2.25E-34	**2.96E-32**
**FITM2**	20q13.12	-0.472	3.29E-34	**4.28E-32**
**PXMP4**	20q11.22	-0.47	8.25E-34	**1.06E-31**
**TGIF2**	20q11.23	-0.469	1.11E-33	**1.40E-31**
**LDLRAD3**	11p13	-0.468	1.38E-33	**1.71E-31**
**NCOA5**	20q13.12	-0.468	1.53E-33	**1.87E-31**
**PAH**	12q23.2	-0.468	1.73E-33	**2.09E-31**
**RALY**	20q11.22	-0.467	1.98E-33	**2.38E-31**
**FARP1**	13q32.2	-0.467	2.18E-33	**2.60E-31**
**RNF43**	17q22	-0.466	3.11E-33	**3.60E-31**
**SLC6A4**	17q11.2	-0.464	5.46E-33	**6.17E-31**
**ATIC**	2q35	-0.462	1.13E-32	**1.25E-30**
**PLCG1**	20q12	-0.461	1.91E-32	**2.06E-30**
**RALGAPB**	20q11.23	-0.461	2.06E-32	**2.19E-30**
**GSS**	20q11.22	-0.459	3.88E-32	**4.04E-30**
**QPRT**	16p11.2	-0.458	4.41E-32	**4.58E-30**
**CSTF1**	20q13.2-q13.31	-0.458	5.83E-32	**5.99E-30**
**PDRG1**	20q11.21	-0.457	6.98E-32	**7.13E-30**
**ATP5F1E**	20q13.32	-0.457	7.03E-32	**7.14E-30**
**SLC22A11**	11q13.1	-0.456	1.13E-31	**1.11E-29**
**TTPAL**	20q13.12	-0.455	1.62E-31	**1.57E-29**
**OSER1**	20q13.12	-0.454	1.64E-31	**1.58E-29**
**SMYD5**	2p13.2	-0.454	1.74E-31	**1.67E-29**
**LOC646762**	7p14.3	-0.454	2.27E-31	**2.15E-29**
**CBFA2T2**	20q11.21-q11.22	-0.453	2.40E-31	**2.26E-29**
**DTNB**	2p23.3	-0.453	2.48E-31	**2.33E-29**
**TSPAN6**	Xq22.1	-0.453	2.82E-31	**2.64E-29**
**CELP**	9q34.13	-0.452	3.41E-31	**3.18E-29**
**SS18L1**	20q13.33	-0.449	1.08E-30	**9.82E-29**
**RUBCNL**	13q14.13	-0.448	1.57E-30	**1.40E-28**
**NDRG3**	20q11.23	-0.448	1.69E-30	**1.49E-28**

**DIDO1**	20q13.33	-0.446	2.53E-30	**2.21E-28**
**LRRC37A3**	17q24.1	-0.446	2.66E-30	**2.31E-28**
**CHMP4B**	20q11.22	-0.445	4.19E-30	**3.57E-28**
**VAV3**	1p13.3	-0.444	5.08E-30	**4.29E-28**
**EPDR1**	7p14.1	-0.444	5.78E-30	**4.84E-28**
**CSNK2A2**	16q21	-0.444	5.80E-30	**4.84E-28**
**GPR143**	Xp22.2	-0.444	6.11E-30	**5.06E-28**
**ERGIC3**	20q11.22	-0.443	8.35E-30	**6.82E-28**
**CSE1L**	20q13.13	-0.441	1.25E-29	**1.01E-27**
**SATB2**	2q33.1	-0.441	1.67E-29	**1.34E-27**
**KIF3B**	20q11.21	-0.44	2.04E-29	**1.62E-27**
**ARFGEF2**	20q13.13	-0.44	2.13E-29	**1.68E-27**
**NCK2**	2q12.2	-0.44	2.27E-29	**1.77E-27**
**CHD6**	20q12	-0.439	2.41E-29	**1.87E-27**
**CIAO1**	2q11.2	-0.439	2.56E-29	**1.98E-27**
**LY6G6E**	6p21.33	-0.439	2.65E-29	**2.04E-27**
**GRPR**	Xp22.2	-0.439	2.99E-29	**2.29E-27**
**POU5F1B**	8q24.21	-0.439	3.04E-29	**2.32E-27**
**SPACA3**	17q11.2	-0.437	5.01E-29	**3.79E-27**
**CHN2**	7p14.3	-0.437	5.04E-29	**3.79E-27**
**STK4**	20q13.12	-0.437	6.11E-29	**4.56E-27**
**ZNF563**	19p13.2	-0.436	7.08E-29	**5.25E-27**
**JADE3**	Xp11.3	-0.435	9.47E-29	**6.95E-27**
**PRDX5**	11q13.1	-0.435	9.61E-29	**7.02E-27**
**REEP1**	2p11.2	-0.435	1.01E-28	**7.37E-27**
**PHACTR3**	20q13.32-q13.33	-0.435	1.02E-28	**7.41E-27**
**LY6G6F**	6p21.33	-0.434	1.26E-28	**9.11E-27**
**UBE2V1**	20q13.13	-0.433	1.70E-28	**1.22E-26**
**ZSWIM1**	20q13.12	-0.433	1.89E-28	**1.35E-26**
**EIF6**	20q11.22	-0.432	2.29E-28	**1.63E-26**
**PSMA7**	20q13.33	-0.431	3.09E-28	**2.18E-26**
**DNMT3B**	20q11.21	-0.429	7.25E-28	**5.04E-26**
**PAAF1**	11q13.4	-0.428	9.01E-28	**6.22E-26**
**EREG**	4q13.3	-0.428	9.83E-28	**6.76E-26**
**SERPINA10**	14q32.13	-0.427	1.11E-27	**7.59E-26**
**WDR35**	2p24.1	-0.427	1.33E-27	**9.01E-26**
**DMD**	Xp21.2-p21.1	-0.426	1.89E-27	**1.27E-25**
**SLC6A6**	3p25.1	-0.425	2.12E-27	**1.42E-25**
**APLF**	2p13.3	-0.425	2.32E-27	**1.54E-25**
**DKC1**	Xq28	-0.424	2.83E-27	**1.87E-25**
**PCMTD2**	20q13.33	-0.424	2.97E-27	**1.95E-25**
**KRBOX4**	Xp11.3	-0.424	3.25E-27	**2.12E-25**
**SYS1**	20q13.12	-0.423	4.32E-27	**2.80E-25**
**NOL4L**	20q11.21	-0.422	6.36E-27	**4.07E-25**
**GGT7**	20q11.22	-0.421	8.05E-27	**5.10E-25**
**LRRC2**	3p21.31	-0.421	8.97E-27	**5.66E-25**
**SPIN3**	Xp11.21	-0.418	2.13E-26	**1.32E-24**
**RPN2**	20q11.23	-0.417	2.82E-26	**1.71E-24**
**MTG2**	20q13.33	-0.416	3.25E-26	**1.97E-24**
**SPATA25**	20q13.12	-0.415	4.90E-26	**2.94E-24**
**MAB21L4**	2q37.3	-0.414	6.73E-26	**3.99E-24**
**LINC02418**	12q24.33	-0.414	6.89E-26	**4.07E-24**
**SLC35C2**	20q13.12	-0.414	7.13E-26	**4.18E-24**
**DSN1**	20q11.23	-0.413	7.79E-26	**4.56E-24**
**NCBP2**	3q29	-0.413	7.83E-26	**4.57E-24**
**ZBTB10**	8q21.13	-0.413	9.14E-26	**5.30E-24**
**PTP4A3**	8q24.3	-0.413	9.72E-26	**5.62E-24**
**NODAL**	10q22.1	-0.413	9.85E-26	**5.67E-24**
**COMMD7**	20q11.21	-0.412	1.05E-25	**6.00E-24**
**PABPC1L**	20q13.12	-0.412	1.10E-25	**6.26E-24**

**DRD2**	11q23.2	-0.412	1.32E-25	**7.43E-24**
**IFT52**	20q13.12	-0.411	1.59E-25	**8.82E-24**
**ZNF251**	8q24.3	-0.41	1.84E-25	**1.02E-23**
**MEX3A**	1q22	-0.41	1.85E-25	**1.02E-23**
**PTPN1**	20q13.13	-0.41	1.97E-25	**1.08E-23**
**TPX2**	20q11.21	-0.41	2.12E-25	**1.16E-23**
**SCML2**	Xp22.13	-0.41	2.13E-25	**1.16E-23**
**F7**	13q34	-0.41	2.14E-25	**1.16E-23**
**ZGPAT**	20q13.33	-0.41	2.20E-25	**1.19E-23**
**ARID3A**	19p13.3	-0.41	2.28E-25	**1.23E-23**
**GDPD5**	11q13.4-q13.5	-0.409	2.62E-25	**1.40E-23**
**ATP9A**	20q13.2	-0.409	2.69E-25	**1.43E-23**
**ZHX3**	20q12	-0.409	2.78E-25	**1.48E-23**
**ACOT8**	20q13.12	-0.408	3.92E-25	**2.05E-23**
**TPD52L2**	20q13.33	-0.408	4.26E-25	**2.22E-23**
**CDX2**	13q12.2	-0.405	9.24E-25	**4.72E-23**
**FAM217B**	20q13.33	-0.405	1.02E-24	**5.19E-23**
**DNTTIP1**	20q13.12	-0.404	1.32E-24	**6.65E-23**
**PCIF1**	20q13.12	-0.403	1.58E-24	**7.87E-23**
**PROSER1**	13q13.3	-0.402	2.32E-24	**1.14E-22**
**GMEB2**	20q13.33	-0.4	3.36E-24	**1.63E-22**
**ZMYND8**	20q13.12	-0.4	3.50E-24	**1.70E-22**
**AMACR**	5p13.2	-0.399	4.66E-24	**2.24E-22**
**SPATA2**	20q13.13	-0.399	5.43E-24	**2.58E-22**
**SNHG17**	20q11.23	-0.398	6.01E-24	**2.83E-22**
**MAP3K20**	2q31.1	-0.398	6.40E-24	**3.01E-22**
**LAS1L**	Xq12	-0.398	6.65E-24	**3.12E-22**
**ETNK2**	1q32.1	-0.397	8.08E-24	**3.76E-22**
**CPN1**	10q24.2	-0.397	9.21E-24	**4.27E-22**
**CAMKV**	3p21.31	-0.397	9.55E-24	**4.41E-22**
**USP27X**	Xp11.23	-0.397	9.67E-24	**4.46E-22**
**UMODL1**	21q22.3	-0.397	9.71E-24	**4.47E-22**
**LOC148709**	1q32.1	-0.396	1.11E-23	**5.09E-22**
**UCKL1**	20q13.33	-0.396	1.28E-23	**5.82E-22**
**ZNF74**	22q11.21	-0.395	1.43E-23	**6.44E-22**
**SNX21**	20q13.12	-0.395	1.44E-23	**6.45E-22**
**AOAH**	7p14.2	-0.394	1.76E-23	**7.78E-22**
**ASCL2**	11p15.5	-0.394	1.97E-23	**8.67E-22**
**SYN3**	22q12.3	-0.394	2.03E-23	**8.95E-22**
**GPSM2**	1p13.3	-0.394	2.28E-23	**1.00E-21**
**NUFIP1**	13q14.12	-0.393	2.62E-23	**1.14E-21**
**ABAT**	16p13.2	-0.392	3.13E-23	**1.36E-21**
**NFE2L3P2**	17q21.32	-0.392	3.16E-23	**1.37E-21**
**SLC2A12**	6q23.2	-0.391	4.61E-23	**1.97E-21**
**SPINDOC**	11q13.1	-0.391	4.62E-23	**1.97E-21**
**TTC25**	17q21.2	-0.39	5.56E-23	**2.35E-21**
**TDGF1**	3p21.31	-0.39	5.77E-23	**2.43E-21**
**ZNF470**	19q13.43	-0.39	6.51E-23	**2.73E-21**
**ANO9**	11p15.5	-0.39	6.80E-23	**2.85E-21**

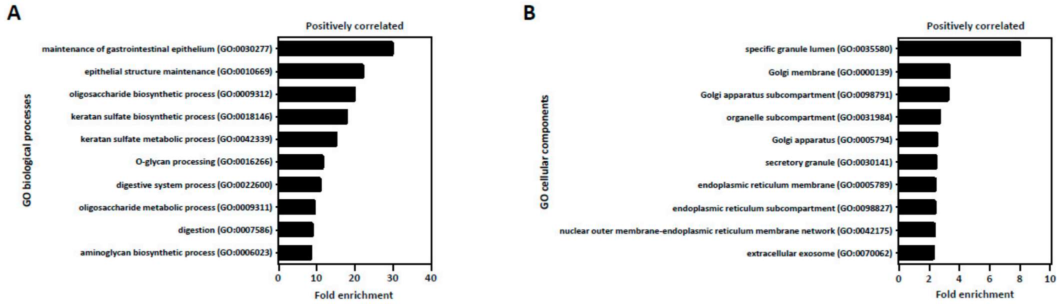

Figure S1. The biological process and cellular component terms enriched with *CTSE* upregulation. The genes that were coexpressed with *CTSE* in CRC from the TCGA database (n = 594) were examined using the cBioPortal online platform (http://cbioportal.org). The top 200 genes co-upregulated with *CTSE* were further analyzed using PANTHER (http://pantherdb.org) in accordance with *(*A*)* biological processes or *(*B*)* cellular components and rated by *fold enrichment* for functional annotation.

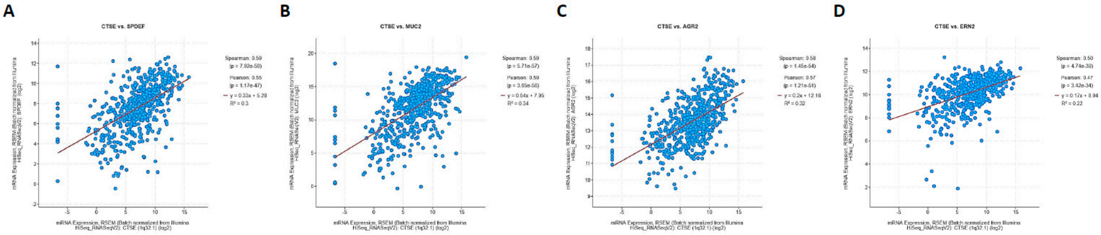

Figure S2. Associations among *CTSE*, *SPDEF*, *MUC2*, *AGR2*, and *ERN2* gene expression. *The data were exported from* the TCGA database (n = 594) using the cBioPortal online platform.

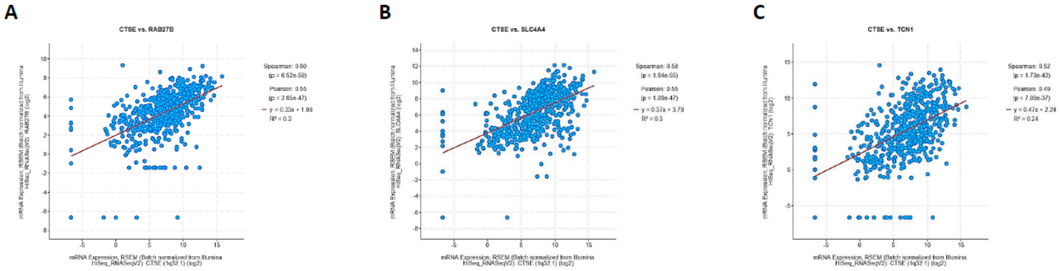

Figure S3. Associations among *CTSE*, *RAB27B*, *SLC4A4*, and *TCN1* gene expression. *The data were exported from* the TCGA database (n = 594) using the cBioPortal online platform.
